# Moss as a Substitute for Linseed Meal and Hot Woolen Cloths1Read before the twenty-seventh annual meeting of the United States Veterinary Medical Association, at Chicago, September 17th, 1890.

**Published:** 1890-10

**Authors:** George H. Berns


### MOSS AS A SUBSTITUTE FOR LINSEED MEAL AND HOT WOOLEN CLOTHS.[1]

BY GEORGE H. BERNS, D.V.M.

PERHAPS there is no class of remedies better known and more frequently used than poultices and fomentations. Their efficacy as remedial agents and their physiological, as well as their therapeutic, actions are so well recognized and understood that it would be a waste of time to even refer to them. There is hardly a day but what all of us order a poultice to a horse's foot, or prescribe hot fomentations to some other part of the body for the purpose of relieving pain and suffering, and it will be admitted that their sphere of usefulness is almost unlimited in the practice of medicine and surgery; yet in very many instances are we obliged to dispense with them, owing to our inability to apply them effectively, for there are many locations on the body of an animal where no poultice and fomentation can be applied or kept.

Linseed meal is practically the only substance used in veter-

[1] Read before the twenty-seventh annual meeting of the United States Veterinary Medical Association, at Chicago, September 17th, 1890.

inary practice to make poultices, and while it is safe, retains heat and moisture well and answers the purpose in the majority of instances, it has many disadvantages.

*First.*—It requires considerable skill and time to prepare and properly apply a linseed meal poultice.

*Second.*—They are bulky and heavy, and tend to sag down from their own weight, and on that account cannot be applied to many parts of the body.

*Third.*—They are rather dirty, and during warm weather are apt to become foul unless very frequently changed, and it requires time and labor to clean the parts after a poultice has been removed.

Hot fomentations are usually made by saturating cloths or woolen blankets in hot water and applying them to the desired parts while hot and covering them with oil silk or some other air-tight material, and in regions where they cannot be kept in position we content ourselves with ordering the parts simply bathed with hot water. That these methods are extremely laborious, troublesome, and, in many instances, inefficient is admitted by all, and I have no doubt that many attempts have been made to improve upon them. Spongio piline is, perhaps, the only substitute that has been offered to the profession as a convenient vehicle to apply heat and moisture, and while it does its work well it is hardly applicable in veterinary practice, on account of its high price and its many other disadvantages. To devise some other means of applying fomentations and poultices than the antiquated methods referred to has occupied considerable of my time and attention for a number of years, and upon trying a variety of substances, I found one which I considered of sufficient value to experiment with for months, and I am pleased to say that my time and labor have not been entirely wasted, for in my own practice I have employed it extensively for a long time and with most satisfactory results.

The substance referred to is a herbaceous plant found in swampy and shady localities in many sections of the United States, and is known as moss. Dr. Muhlenberg, of Lancaster, Penna., in 1827, describes over 170 varieties, all of which possess the power of imbibing and retaining large quantities of moisture, and seem to have a peculiar resistance against the actions of ferments, for I have never known moss to become foul or offensive, no matter how long or in what way employed. Some of the mosses are extremely coarse, with woody stems and ill-developed, short leaves. The plant I have adopted as a substitute for oil meal and woolen cloths is a very fine, long thin-stemmed vegetable, with soft, slender, long stems and branches, well coated with large serrated leaves. Carefully conducted experiments have proven that this variety, at least, is positively non-irritating, even to the most delicate and sensitive surfaces, but that it possesses properties which I believe will in time give it a prominent place among the remedial agents of the U. S. Pharmacopœia.

*First.*—Moss possesses the power of retaining moisture for a longer period of time than any other substances known, and if properly protected by air-tight coverings, it will retain heat fully as long, if not longer, than ground flaxseed.

*Second.*—If applied to the body in a proper manner, it can be kept saturated without removal or reapplication, thus avoiding a great deal of time and labor to attendant, risk of exposure and annoyance to patient.

*Third.*—It can be rendered antiseptic, astringent, anodyne or irritating as desired, by saturating with such solutions, and affords a most convenient and efficacious means of dressing wounds.

*Fourth.*—It is soft, pliable and suitably elastic, and will adapt itself to almost any position where no poultice or surgical dressing can be applied.

*Fifth.*—It is cleanly, cheap, and if properly prepared extremely easy of application. Recognizing these properties and realizing the difficulties most always met with in placing and retaining in position poultices, fomentations and surgical dressings, I conceived the idea of placing the moss into sacks or bags, quilting or tufting them to guard against displacement, and making them into shapes and sizes to fit most any part of the body. After months of experimenting, I am at last in a position to place before the veterinary profession a complete set of moss fomentation pads, which have in my practice proven to afford a most convenient and efficacious means of applying fomentations or surgical dressings. The result of our labors in this direction has been the production of thirteen different styles and shapes of moss pads, intended to cover those parts of the body that most frequently require surgical attention.

I will not tire your patience by giving a minute description of all of them, but would beg your indulgence for a few moments longer to say a few words upon one or two of them. One of the most useful of the thirteen is probably the foot pad. It is made in the shape of the foot and from 1¼ to 1½ inches in thickness, saturated in water or any medicated solution that may be indicated and placed under the foot of a horse; kept in place like any other poultice it will adapt itself immediately to the irregularities of the plantar surface, and afford an equal distribution of pressure to the parts, thus preventing in a great measure the formation of exuberant granulations so troublesome in all cases where it becomes necessary to remove portions of the horny sole. It forms a soft and elastic cushion and means of rest, and in laminitis it affords ease and comfort at once; it will remain where placed, is perfectly clean and easily resaturated without removal by simply dipping the foot into hot water or any medicated solution, which it absorbs quickly and afterwards retains. In my practice the use of linseed poultices for foot troubles has been almost entirely abandoned, for the moss pad is cleaner, and, in my experience, far more efficacious. The pastern and ankle pads are next to the foot pads most frequently used, and that they will adapt themselves to the conformation of the parts and allow an equal distribution of pressure is demonstrated by a few specimens that have been used and allowed to dry on a horse's leg.

